# System metabolic engineering modification of *Saccharomyces cerevisiae* to increase SAM production

**DOI:** 10.1186/s40643-025-00858-9

**Published:** 2025-03-12

**Authors:** Liangzhuang Tan, Yuehan Zhang, Ping Liu, Yihang Wu, Zuoyu Huang, Zhongce Hu, Zhiqiang Liu, Yuanshan Wang, Yuguo Zheng

**Affiliations:** 1 Key Laboratory of Bioorganic Synthesis of Zhejiang Province, College of Biotechnology and Bioengineering, Zhejiang University of Technology, No. 18, Chaowang Road, Hangzhou, Zhejiang Province 310014 P. R. China; 2 Engineering Research Centre of Bioconversion and Biopurification, Ministry of Education, Zhejiang University of Technology, No. 18, Chaowang Road, Hangzhou, Zhejiang Province 310014 P. R. China; 3 The National and Local Joint Engineering Research Centre for Biomanufacturing of Chiral Chemicals, Zhejiang University of Technology, Hangzhou, 310014 P. R. China

**Keywords:** *S*-adenosyl-L-Methionine, *Saccharomyces cerevisiae*, CRISPR/Cas9 system, Combinatorial metabolic engineering, Fed-batch fermentation, TCA cycle

## Abstract

S-adenosyl-L-methionine (SAM) is an important compound with significant pharmaceutical and nutraceutical applications. Currently, microbial fermentation is dominant in SAM production, which remains challenging due to its complex biosynthetic pathway and insufficient precursor availability. In this study, a multimodule engineering strategy based on CRISPR/Cas9 was established to improve the SAM productivity of *Saccharomyces cerevisiae*. This strategy consists of (1) improving the growth of *S. cerevisiae* by overexpressing the *hxk2* gene; (2) enhancing the metabolic flux toward SAM synthesis by upregulating the expression of the *aat1, met17*, and *sam2* genes and weakening the synthesis pathway of L-threonine; (3) elevating precursor ATP synthesis by introducing the *vgb* gene; (4) blocking the SAM degradation pathway by knocking out the *sah1* and *spe2* genes. The SAM titer of the resulting mutant AU18 reached 1.87 g/L, representing an increase of 227.67% compared to the parental strain. With optimal medium, SAM titer of mutant AU18 reached 2.46 g/L in flask shake fermentation. The SAM titer of mutant AU18 further reached 13.96 g/L after 96 h incubation with a continuous L-Met feeding strategy in a 5 L fermenter. Therefore, with comprehensive optimization of both synthesis and degradation pathways of SAM, a multimodule strategy was established, which significantly elevated the SAM production of *S. cerevisiae*. This laid a foundation for the construction of hyperproducer for SAM and other valuable amino acids or chemicals.

**Supplementary Information:**

The online version contains supplementary material available at 10.1186/s40643-025-00858-9.

## Graphical Abstract

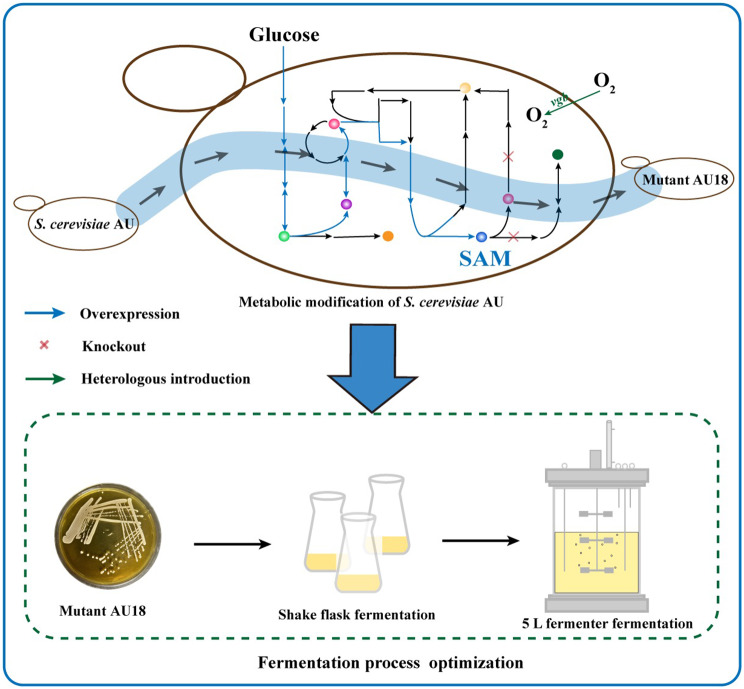

## Introduction

Ubiquitous presents in all organisms, *S*-adenosyl-L-methionine (SAM) is the second most abundant cofactor in nature. It plays a critical role in methylation, sulfurylation, and aminopropylation processes (Darroudi and Ziarani 2021; Huang et al. 2022; Pietrocola et al. 2019; Xuan et al. 2024). SAM exhibits significant efficacy in the treatment of diseases such as liver disease (Bian et al. 2021), depression (Girone et al. 2023), and cancer (Mathes et al. 2024). It has been also approved as a prescription dietary supplement (Chu et al. 2013). Therefore, the demand for SAM increases rapidly. Currently, as an environment benign, cost-effective, and sustainable method, microbial fermentation is dominant in SAM production compared chemical synthesis and enzymatic catalysis (Mamontova et al. 2018; Ravi et al. 2014; Wang et al. 2024a; Yu and Zhu 2017).

Due to its high SAM accumulation capacity and safety (classified as generally recognized as safe organism by Food and Drug Administration of USA) (Lian et al. 2018), *Saccharomyces cerevisiae* is widely used for SAM production. Various metabolic engineering strategies have been employed to enhance SAM titer in *S. cerevisiae*, including the improvement of biosynthetic pathways, the weakening of competing pathways, and the disruption of degradation pathways (Li et al. 2023). The details of these strategies are as follows: (1) Optimizing the synthesis pathway of SAM. This involves not only enhancing the SAM synthesis pathways but also attenuating competing pathways. Chen et al. (2016a) enhanced the SAM biosynthesis pathway in *S. cerevisiae* CGMCC2842 by co-expressing *met6* and *sam2* genes. The SAM titer of the resulting engineered strain YGSPM reached 1.55 g/L, increased by 234% compared to the parental strain. Chen et al. (2016b) also blocked the GYC (glyoxylate cycle) branch pathway by disrupting *mls1* gene and enhanced conversion of acetic acid to acetyl-CoA by overexpressing *acs2* gene in *S. cerevisiae* 2842. SAM titer of the resulting mutant Y*mls1*∆ reached 1.52 g/L. Fu et al. (2023) mutated the enzyme encoded by the *sam2* gene and generated the variant SAM2^S203F, W164R, T251S, Y285F, S365R^. This variant exhibited almost 2-fold higher catalytic activity as the parental enzyme and was subsequently introduced into the *S. cerevisiae* strain BY4741. The SAM titer of the recombinant strain BSM7 reached 613.52 mg/L, which was 2.9-fold higher than that of the control. With further pathway optimization (knocking out the *sah1* and *glc3* genes) and fermentation process optimization, the SAM titer of the resulting mutant BSM8 in shake-flask fermentation reached 1.25 g/L, which was 7-fold higher than that of the control. (2) Increasing ATP supply: ATP plays a crucial role in biosynthesis, metabolic regulation, and cellular homeostasis. In addition, ATP serves as both a precursor and an energy donor for SAM synthesis (Chen 2020; Man et al. 2020). Therefore, enhancing ATP availability is essential for improving SAM production. Wang et al. (2024b) enhanced the acetyl-CoA supply of *S. cerevisiae* by knocking out the *mls1* gene, resulting into an increase in ATP availability and an 8.92% increase in SAM titer of the resulting engineered strain. Hu et al. (2023) enhanced the respiratory activity of *S. cerevisiae* by knocking out the Cu/Zn superoxide dismutase encoding gene *sod1*, resulting in a 22.3% increase in SAM titer of the resulting engineered strain. (3) Blocking the catabolic pathway of SAM: Because of SAM is the primary methyl donor in living organisms, disrupting methylation reactions may promote the accumulation of SAM. The 24-sterol C-methyltransferase (encoded by *erg6*) catalyzes the formation of SAH (*S*-adenosyl-L-homocysteine) from SAM in *S. cerevisiae*. Xiao et al. (2024) knocked out *erg6* gene in *S. cerevisiae* and resulted in a 10.39% increase in SAM titer of the resulting engineered strain C26P. Wang et al. (2024b) disrupted the *sah1* gene of mutant SC04, led to the excessive accumulation of SAH, thereby enhanced feedback inhibition and reduced further metabolism of SAM. The SAM titer of mutant SC05 reached 1.55 g/L, resulting in a 313.64% increase compared to the parental strain.

In addition to metabolic modification, mutagenesis breeding is also an important strategy for enhancing SAM production. Hu et al. (2024) obtained a mutant 616-19-5 through UV (ultraviolet) mutagenesis in conjunction with nystatin/sinefungin and high-throughput screening. The SAM titer of mutant 616-19-5 reached 1.39 g/L, resulting in a 329% increase compared to the parental strain. Furthermore, Weng et al. (2022) obtained a high SAM producing mutant T11-1 utilizing a combination of ARTP (atmospheric and room temperature plasma) and UV-LiCl mutagenesis, followed by droplet microfluidic cultivation (with ethionine, L-Met, nystatin and cordycepin as screening agents). The SAM titer of mutant T11-1 reached 10.72 g/L in a 5 L fermenter. Huang et al. (2012) obtained a mutant H5M147 by space mutagenesis of *S. cerevisiae* H5 and further constructed an engineered mutant H5MR83 by integrating *sam2* gene into the genome of H5M147. The SAM titer of mutant H5MR83 reached 9.64 g/L in a 50 L fermenter, resulting in a 537% increase compared to the parental strain H5. Furthermore, it is well known that the components of fermentation medium play a significant role in the growth of the strain and the synthesis of products. Therefore, medium optimization is an effective approach to enhance SAM production. Fu et al. (2023) optimized the fermentation medium components using an orthogonal experimental strategy and elevated the SAM titer of strain BSM8 from 0.66 to 0.78 g/L in shake flask fermentation. Xiao et al. (2024) optimized the L-Met feeding strategy using a single-factor approach and elevated the SAM titer of strain C262P6S from 1.55 to 1.77 g/L. A Bayesian optimization strategy was further applied to optimize the medium composition and elevated the SAM titer to 2.97 g/L in shake flask fermentation. Wang et al. (2024b) used a single-factor experiment to optimize both the medium composition and L-Met feeding strategy, which increased the SAM titer of strain SC06 from 0.24 to 0.47 g/L in shake flask fermentation.

Despite these advancements (Table 1), microbial production of SAM still faces challenges. The metabolic pathway of SAM in *S. cerevisiae* is extremely sophisticated, often necessitating enhancement of primary biosynthetic routes. However, investigations on enhancing the synthesis pathway of SAM mainly focus on the metabolic pathway from L-aspartic acid to SAM, while neglecting the crucial node where this pathway competes with the TCA (tricarboxylic acid) cycle for oxaloacetate. Furthermore, modifications to competitive pathways have mainly focused on weakening the L-cystathionine branch with limited attention given to other branches, such as the ornithine and L-threonine pathways. In addition, the supply of ATP is a bottlenecking factor for the synthesis of SAM, while the introduction of *Vitreoscilla* hemoglobin (VHb, encoded by *vgb*) has been shown to increase intracellular oxygen level and promote ATP synthesis (Stark et al. 2012). However, introduction of *vgb* gene into *S. cerevisiae*, which might improve the SAM synthesis efficiency, has been seldomly investigated.

**Fig. 1:**
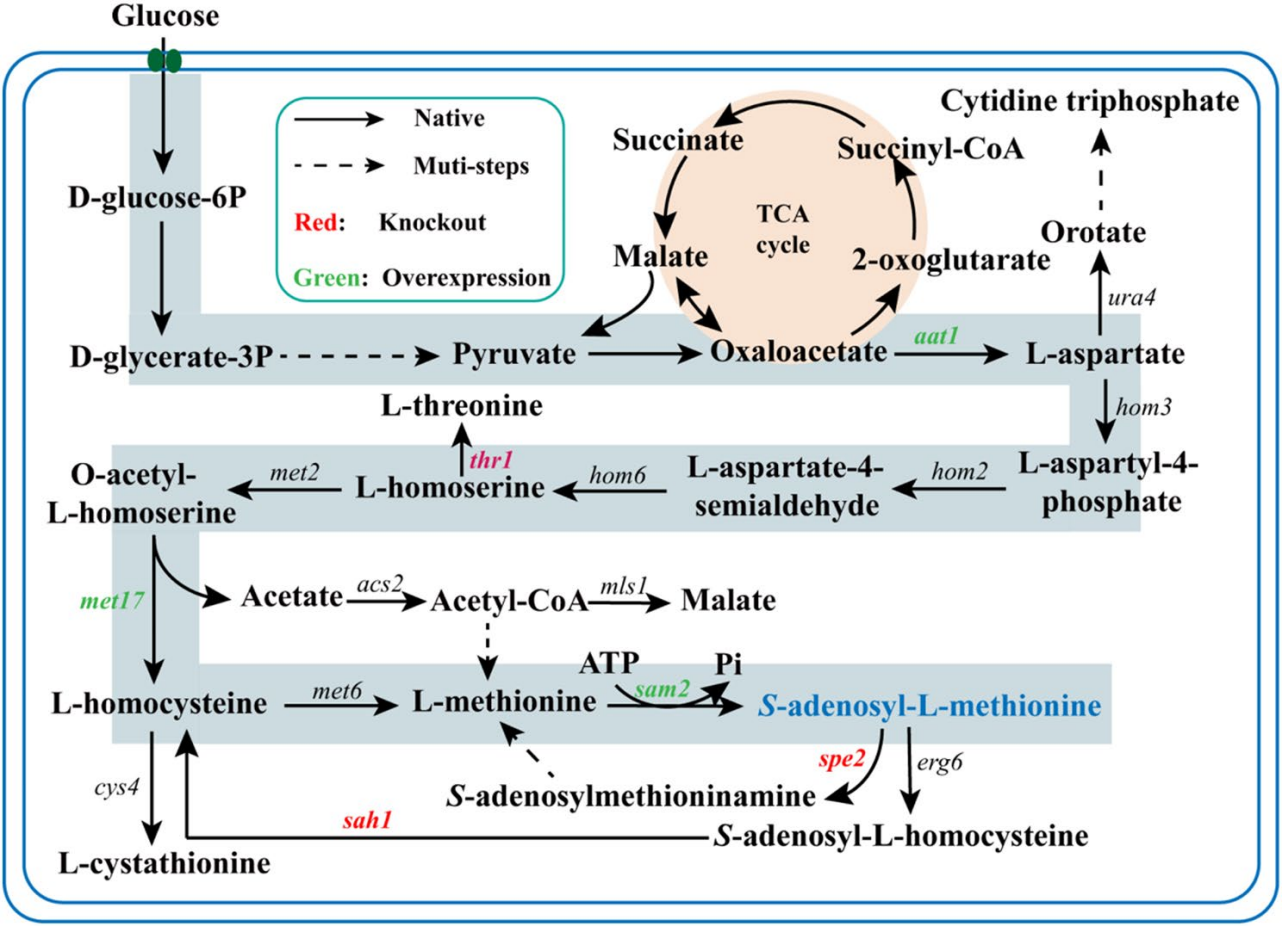
SAM biosynthetic pathway from glucose in *S. cerevisiae*. The orange background represents TCA cycle, while the blue background indicates the steps of SAM biosynthesis. Black solid arrows indicate native pathways, dashed arrows denote multistep reactions, red font arrows show gene knockout, and green font arrows represent gene overexpression

In this study, several metabolic engineering strategies were employed to enhance SAM production in *S. cerevisiae* AU (Fig. 1). The *hxk2* gene was firstly overexpressed to improve growth of the strain. Subsequently, the L-threonine branch pathway was weakened, and the SAM synthesis pathway was strengthened to redirect carbon flux toward SAM biosynthesis. Additionally, the *vgb* gene was introduced to improve ATP supply. To further enhance SAM accumulation, the SAM degradation pathways were disrupted by knocking out the *spe2* and *sah1* genes. Finally, the shake flask fermentation medium was optimized to assess the nutritional preferences of the mutant AU18, and its performance was subsequently verified in a 5 L fermenter. This study systematically manipulated the metabolic pathways involved in SAM biosynthesis in *S. cerevisiae*, providing a foundation for the development of a hyperproducer of SAM.

**Table 1:** Research progress on the synthesis of SAM in *S. cerevisiae* over the past five years

Strains	Strategies	Titer (g/L)	Source
*S. cerevisiae* 616-4-7	UV mutagenesis	10.92	(Hu et al. 2024)
*S. cerevisiae SC06*	Knocking out *lsc2* and *mls1*	1.25	(Wang et al. 2024b)
*S. cerevisiae C262P6S*	Combinatorial metabolic engineering and Bayesian optimization	2.97	(Xiao et al. 2024)
*S. cerevisiae* WT15-33	Knocking out *sod1* and optimization of fermentation process	10.1	(Hu et al. 2023)
*S. cerevisiae* ZY1-5	ARTP and UV-LiCl mutagenesis, optimization of fermentation process	10.72	(Weng et al. 2022)
*S. cerevisiae* CGMCC 2842	Knocking out *reg1* and overexpressing *snf1*	8.28	(Chen et al. 2022)
*S. cerevisiae* BY4742	Overexpressing *snz3, rfc4*, and *rps18b*	0.9	(Dong et al. 2021)
*S. cerevisiae* CGMCC 2842	Knocking out *kcs1* and *arg82*	8.86	(Chen et al. 2021)
*S. cerevisiae* CGMCC 13,760	Optimization of fermentation medium	16.14	(Li et al. 2020)
*S. cerevisiae* AU18	System metabolic engineering modification and optimization of fermentation process	13.98	This study

## Materials and methods

### Strains and culture conditions

The mutant *S. cerevisiae* AU was maintained in our laboratory and served as the parental strain for further genome editing. *E. coli* DH5α was used as the cloning host for plasmid and fragment construction. Details of all strains used in this study were listed in Table 2. *S. cerevisiae* was incubated in YPD (yeast peptone dextrose) agar (20 g/L glucose, 20 g/L peptone, 10 g/L yeast extract, 20 g/L agar) plate at 30 ℃ for 48 h. Single colonies were inoculated into test tubes containing 5 mL of YPD medium and incubate at 30 ℃ and 220 rpm for 12 h. *E. coli* DH5α was incubated in LB (Luria Bertani) medium (10 g/L tryptone, 10 g/L NaCl, and 5 g/L yeast extract) at 37 ℃ and 220 rpm for 8 h.

**Table 2:** Strains used or constructed in this study

Strains	Descriptions	Source
*E. coli* DH5α	Cloning host	Tsingke, China
AU	Starting strain *S. cerevisiae*	Lab collection
AU01	AU harboring plasmid pESC-*hxk2*	This study
AU02	AU01 deleting *ura4*	This study
AU03	AU01, Δ*thr1*∷P_TEF_-*hxk2*-T_TEF_	This study
AU04	AU01 deleting *cys4*	This study
AU05	AU03 harboring plasmid pESC*-aat1*	This study
AU06	AU03 harboring plasmid pESC*-hom3*	This study
AU07	AU03 harboring plasmid pESC- *hom2*	This study
AU08	AU03 harboring plasmid pESC- *hom6*	This study
AU09	AU03 harboring plasmid pESC-*met2*	This study
AU10	AU03 harboring plasmid pESC-*met 17*	This study
AU11	AU03 harboring plasmid pESC- *met6*	This study
AU12	AU03 harboring plasmid pESC-*sam2*	This study
AU13	AU03, Δ*Gal80*∷P_TEF_-*sam2*-T_TEF_-P_TEF_-*met17*-T_TEF_-T_TEF_-P_TEF_-*aat1*-T_TEF_	This study
AU14	AU13, Δ*thr1*∷P_TEF_-*vgb*-T_TEF_	This study
AU15	AU14, Δ*erg6*	This study
AU16	AU14, Δ*sah1*	This study
AU17	AU14, Δ*spe2*	This study
AU18	AU14, Δ*sah1*∷P_TEF_-*vgb*-T_TEF_, Δ*spe2*∷P_TEF_-*aat1*-T_TEF_	This study

## Knocking out genes

The CRISPR/Cas9 gene editing system (containing Cas9, sgRNA, and donor DNA) was used to induce gene deletion and substitutive mutations in the strains (Laughery and Wyrick 2019). Here, the process of gene *thr1* deletion was presented as an example. Construction of sgRNA: Firstly, the PAM site of the *thr1* gene was identified using the Benchling website (https://www.benchling.com/). The primers were designed using SnapGene software and the PAM site in the sgRNA was replaced by PCR. The PCR product was then transformed into *E. coli* DH5α, which were plated onto LB agar plates supplemented with 100 µg/mL ampicillin. After 14 h of incubation, positive sgRNAs were identified by colony PCR and sequencing. Construction of donor DNA: Homologous arms (500 bp) on either side of the *thr1* gene were amplified based on the gene sequence, and these fragments were then fused to create the donor-*thr1* (1000 bp) (Fig. S1). Subsequently, the Cas9, sgRNA, and donor-*thr1* were transformed into *S. cerevisiae* via the LiAc/SS carrier DNA/PEG transformation method (Gietz 2014). The transformed cells were plated onto YPD agar plates containing G418 (500 µg/mL) and Hygromycin B (300 µg/mL) to screen positive transformants. Then colony PCR with validation primers were performed to screen *thr1* gene knocking out strains. A list of all primers used in this study is provided in Table S1 of the supporting information.

## Overexpressing genes

Gene overexpression can be achieved through plasmid-based overexpression and integration of the target gene into the host strain. Using the overexpression of *sam2* as example, the specific steps for both methods were described as follows: (1) For plasmid-based overexpression: The *sam2* gene was first amplified from the genome of *S. cerevisiae* BY4741. The amplified *sam2* fragment was then ligated into a linearized pESC plasmid using the ClonExpress II One Step Cloning Kit (Vazyme Biotech Co., Ltd., China). This ligation mixture was transformed into *E. coli* DH5α to construct the pESC-TEF-*sam2* plasmid (Fig. S2). The resulting plasmid was subsequently transformed into *S. cerevisiae* via the LiAc/SS carrier DNA/PEG method (Gietz 2014). The transformed cells were plated onto YPD agar plates containing 300 µg/mL Hygromycin B to select positive transformants. (2) For genomic integration: The method used to integrate the *sam2* gene into the host strain is the same as that used for gene knockout. The only difference is that when constructing the donor DNA, the *sam2* fragment needs to be fused into the middle of the homologous arm of the insertion site.

## Real-time fluorescence quantitative PCR analysis

RNA extraction and reverse transcription were performed using Bacterial RNA extraction kit R403 and HiScript II Q RT SuperMix, respectively, (Vazyme Biotech Co., Ltd.). The system for real-time qPCR is ChamQ Universal SYBR qPCR Master Mix (Vazyme Biotech Co., Ltd.). The reaction procedure of qPCR involved initial denaturation at 95 °C for 30 s, followed by 40 cycles, each cycle consisting of 10 s at 95 ° C and 15 s at 60 ° C. The mRNA level was analyzed with housekeeping gene *act1* as internal reference and calculated by the 2^−ΔCt^ method (ΔCt = Ct (test) - Ct (*act1*) (Pang et al. 2024).

## Glucose determination

Glucose in the fermentation broth was measured using the DNS method (Hagishita et al. 1996). 1 mL fermentation broth was centrifuged at 12,000 rpm for 2 min, then 200 µL supernatant was mixed with 400 µL of DNS solution. The mixture was boiled in water for 5 min, followed by the addition of 1.4 mL of dH_2_O. After thoroughly mixing, the absorbance of the solution at 540 nm (OD_540_) was measured (Wang et al. 2024b).

### SAM production in shake flask fermentation and 5 L fermenter fermentation

For shake flask fermentation, the mutants were grown in test tubes containing 5mL YPD medium at 37 °C and 220 rpm for 12 h to prepare primary seed. The seed was then inoculated (6% v/v) into 250 mL shake flasks containing 50 mL shake flask fermentation medium (30 g/L glucose, 10 g/L peptone, 5 g/L yeast extract, 4 g/L KH_2_PO_4_, 2 g/L K_2_HPO_4_, and 0.5 g/L MgSO_4_·H_2_O), and cultured for 24 h at 30 °C and 220 rpm. Then 2 mL 50 g/L L-Met was supplemented and cultured for another 24 h.

For 5 L fermenter fermentation, 300 mL seed culture was prepared in six 250 ml shake flasks containing 50 mL YPD medium, and the flasks were incubated for 12 h at 30 °C and 220 rpm. Then the seed culture were inoculated into a 5 L fermenter (BIOTECH-5BG, Shanghai Baoxing Bio-Engineering Equipment Co., Ltd, China) containing 2.7 L fermentation medium (20 g/L glucose, 10 g/L yeast extract, 25 g/L peptone, 4 g/L KH_2_PO_4_, 2 g/L K_2_HPO_4_, 0.1 g/L MnSO_4_·7H_2_O, 0.6 g/L ZnSO_4_·7H_2_O, 0.5 g/L MgSO_4_, 0.5 g/L CaCl_2_, 1.6 mg/L CuSO_4_, 4.8 mg/L ammonium molybdate, 0.5 g/L NaCl, 0. 3 mg/L biotin, 3.6 mg/L calcium pantothenate, 3.6 mg/L vitamin B1, 3.6 mg/L vitamin B6, 0.4 g/L sodium citrate). The fermentation was conducted at 30 °C with 10–20% DO (dissolved oxygen) and 500 rpm. The pH was maintained at 5.5 by automatically feeding 40% NH_3_·H_2_O. The glucose was maintained at approximately 1–4 g/L by supplementing the feeding broth (500 g/L glucose and 12 g/L yeast extract). When biomass was stable (about 36 h), 8 g L-Met was supplemented every 4 h (Weng et al. 2022).

## SAM, ATP, and L-Met analysis

For SAM analysis, 1 mL fermentation broth was centrifuged at 12,000 rpm for 2 min. The supernatant was discarded. Then 200 µL ethyl acetate and 200 µL dH_2_O were added to the resulting cells and thoroughly mixed. The mixture was then incubated in a metal bath at 35 °C and 1500 rpm for 30 min. Subsequently, 500 µL of 0.35 mol/L H_2_SO_4_ was added, and the reaction continued for 1.5 h. After the reaction, the mixture was centrifuged at 12,000 rpm for 3 min. The supernatant was then filtered through a 0.22 μm membrane and quantified using HPLC (high-performance liquid chromatography) (Agilent 1260 HPLC system, Agilent, USA) with an UV detector (wavelength 254 nm) and a C18 column (4.6 mm × 250 mm × 5 μm, Welch Materials (Shanghai) Co., Ltd, China). The mobile phase, a mixture of 82% (v/v) salt solution (2 mmol/L sodium 1-heptanesulfonate and 40 mmol/L NH_4_H_2_PO_4_), and 18% (v/v) methanol solution, was run at 1 mL/min at 30 °C (Weng et al. 2022).

For L-Met analysis, 1 mL fermentation broth was centrifuged at 12, 000 rpm for 2 min. The supernatant was discarded, and 2 mL 1.5 mol/L perchloric acid was added and thoroughly mixed. The mixture was then incubated in a metal bath at 30 °C and 1500 rpm for 2 h. HPLC analysis was performed using a mobile phase consisting of 10% methanol and 90% H_2_O at 210 nm. All other conditions were consistent with those used for SAM detection (Wang et al. 2024b).

For intracellular ATP analysis, the samples were treated as described for L-Met analysis, except that the reaction temperature was adjusted to 40 °C. For HPLC analysis, the mobile phase consisted of a mixture of 95% 50 mmol/L phosphate buffer (pH 6.0, containing 1 mmol/L EDTA) and 5% methanol. All other detection conditions were identical to those used for SAM analysis (Wang et al. 2024b).

## Results and discussions

### *hxk2* overexpression improved growth and SAM titer of *S. cerevisiae*

In *S. cerevisiae* metabolism, glucose serves not only as a direct energy source but also as the carbon backbone for the synthesis of biomacromolecules (Olivares-Marin et al. 2018). Therefore, promoting cellular glucose uptake and utilization can theoretically enhance the growth and SAM production of *S. cerevisiae*. Hexokinase isoenzyme 2 (encoded by *hxk2*) is primarily localized in the cytoplasm and plays a crucial role in glucose uptake. This enzyme is also essential for the phosphorylation of intracellular glucose and functions as a critical enzyme in the glycolytic pathway (Gancedo 1998; Rodríguez et al. 2001).

**Fig. 2:**
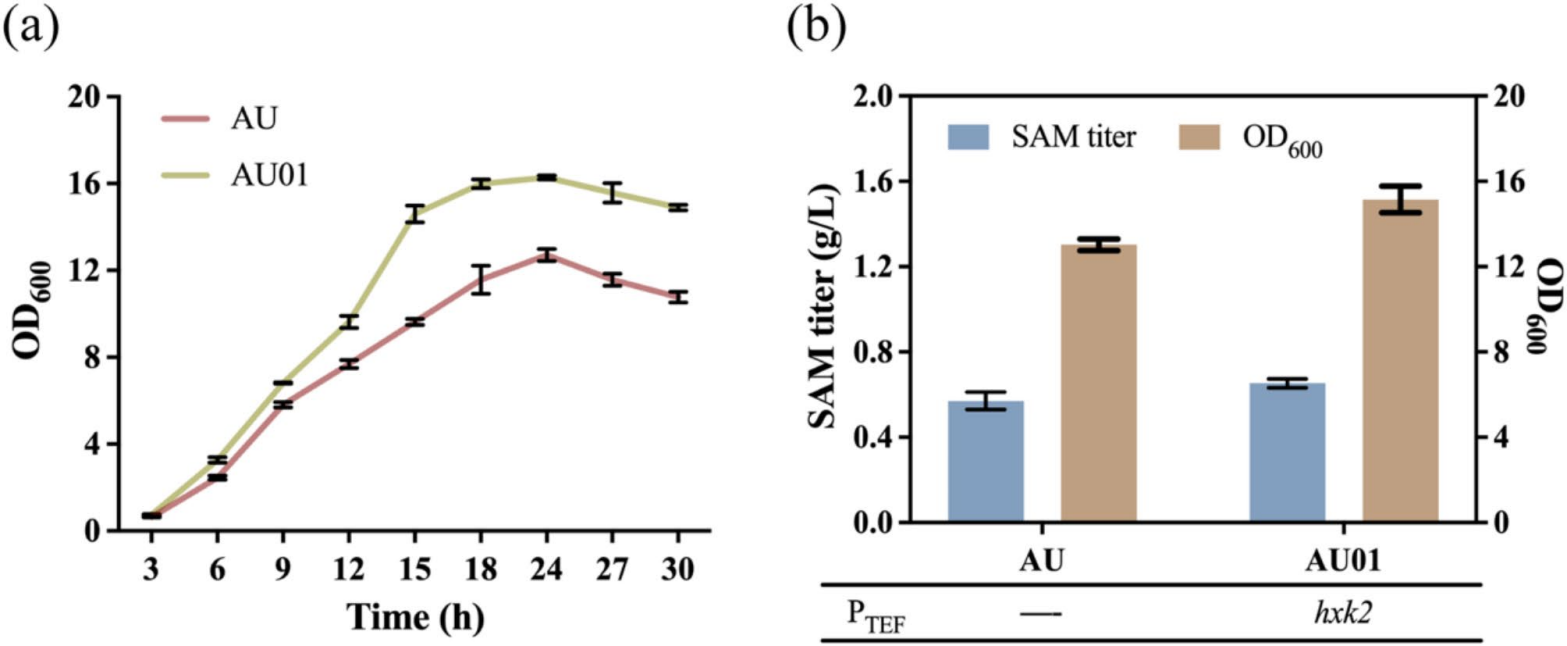
The effect of gene overexpression on growth and SAM titer of the mutants. (**a**) Growth curve of mutant overexpressing *hxk2* gene. (**b**) The SAM titer and growth of mutant overexpressing *hxk2* gene

To evaluate the effect of *hxk2* overexpression on growth and SAM production in *S. cerevisiae*, a mutant (AU01) overexpressing *hxk2* was constructed. The growth of AU01 was monitored by measuring OD_600_ of the fermentation broth. The results showed a significant increase in cell growth of mutant AU01 (Fig. 2a). Subsequently, shake flask fermentation was carried out, and the SAM titer and OD_600_ of AU01 reached 0.65 g/L and 15.15, respectively, resulting into a 16.27% and 11.48% increase compared to the parental strain AU (Fig. 2b). This result may be due to the overexpression of *hxk2* gene, which promotes the fructose phosphorylation reaction in glycolysis, enhances glucose utilization and optimizes metabolism (Pérez et al. 2014), ultimately increases biomass and SAM titer of the strain. Therefore, the *hxk2* gene was integrated into the genome of the mutant in subsequent experiments to further enhance SAM production.

### Weakening competitive pathways

**Fig. 3:**
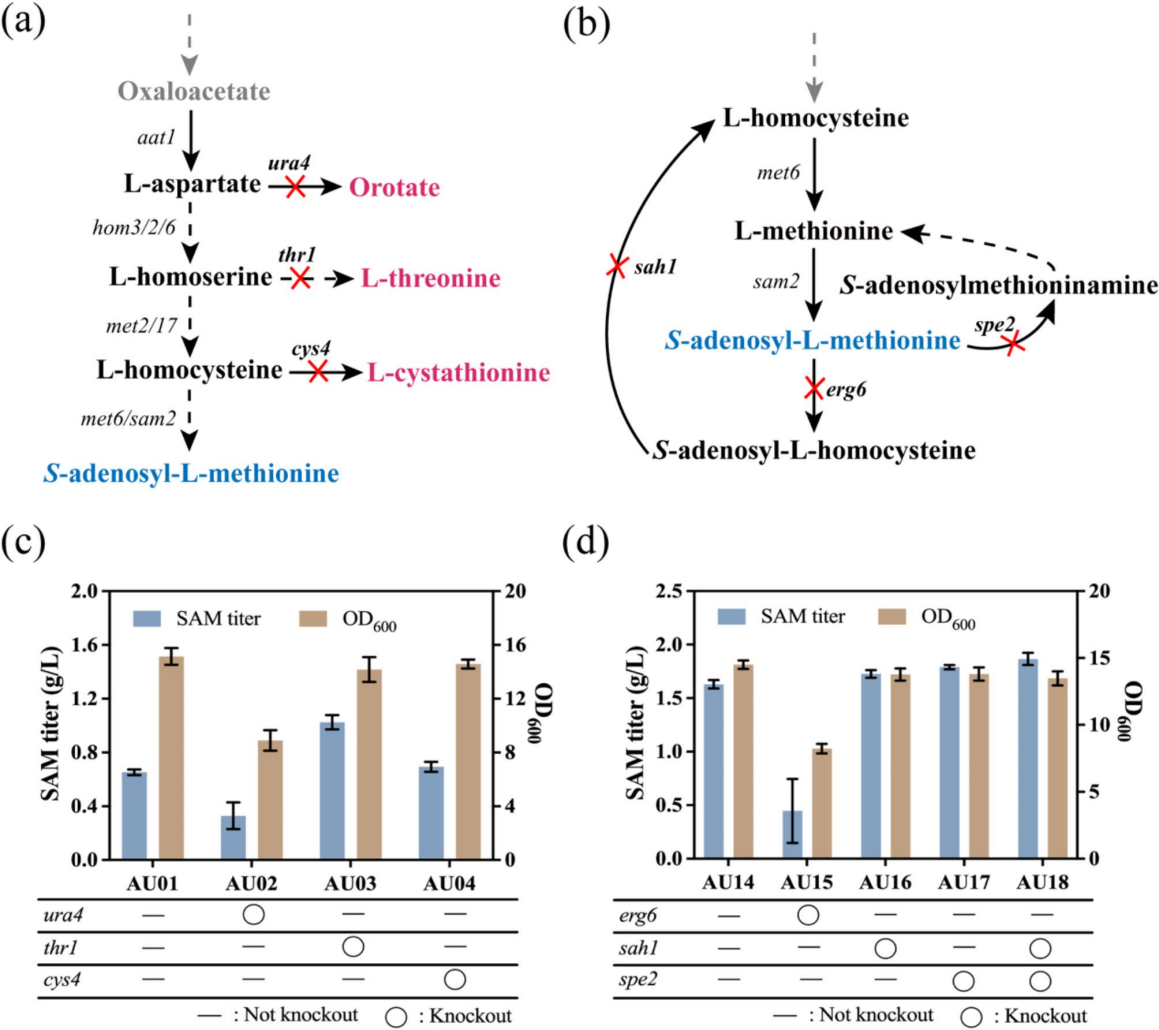
Effect of weakening the competitive pathways and catabolic pathways on SAM production. (**a**) Knocking out key genes in the competitive pathways of SAM synthesis. (**b**) Knocking out key genes in the catabolic pathways of SAM synthesis. (**c**, **d**) SAM titer and growth of mutants in shaking flask fermentation

Redirecting carbon flux away from competing pathways to enhance the titer of target products is a widely used strategy (Choi et al. 2019). The synthesis pathways of orotate, L-threonine, and L-cystathionine compete with the biosynthesis of SAM. Previous studies have successfully developed high SAM-producing strains by deleting key genes involved in the L-cystathionine biosynthesis pathway (Qin et al. 2020). In this study, to direct more carbon flux toward SAM synthesis, several genes involved in the orotate, L-threonine, and L-cystathionine biosynthesis pathways (*ura4, thr1*, and *cys4*, respectively) were knocked out, resulting in the mutants AU02, AU03, and AU04 (Fig. 3a).

The shake flask fermentation results of these mutants showed that only knocking out the *thr1* gene increased SAM titer (Fig. 3c). Specifically, the mutant AU02 produced 0.33 g/L SAM, the mutant AU03 produced 1.03 g/L SAM, resulting in a 58.46% increase compared to mutant AU01, the mutant AU04 produced 0.69 g/L SAM. These findings suggested that reasonable regulation of competitive pathways can improve SAM production. Although L-threonine is an essential amino acid for *S. cerevisiae*, no significant difference in growth of mutant AU03 and AU01 was observed (Fig. 3c). This is likely due to the sufficient supply of L-threonine from the yeast extract (Tao et al. 2023) in the medium, which meets the metabolic demands of mutant AU03. Therefore, the L-threonine synthesis pathway can be blocked to prevent competition for carbon flux with the SAM biosynthesis pathway. However, the knockout of *ura4* gene in the orotate biosynthesis pathway significantly inhibited strain growth and notably reduced SAM synthesis. This can be attributed to the role of orotate as a precursor in the synthesis of purine and pyrimidine nucleotides. The depletion of orotate severely impairs nucleic acid synthesis in *S. cerevisiae* and thus negatively affects growth (Löffler et al. 2016). Additionally, the block of the L-cystathionine biosynthesis pathway did not result in a significant increase in SAM production as reported in previous studies (He et al. 2006; Qin et al. 2020). This suggests that the L-cystathionine biosynthesis pathway is not a major pathway which competes for carbon flux with SAM in this engineered strain.

### Strengthening the synthetic pathway of SAM

**Fig. 4:**
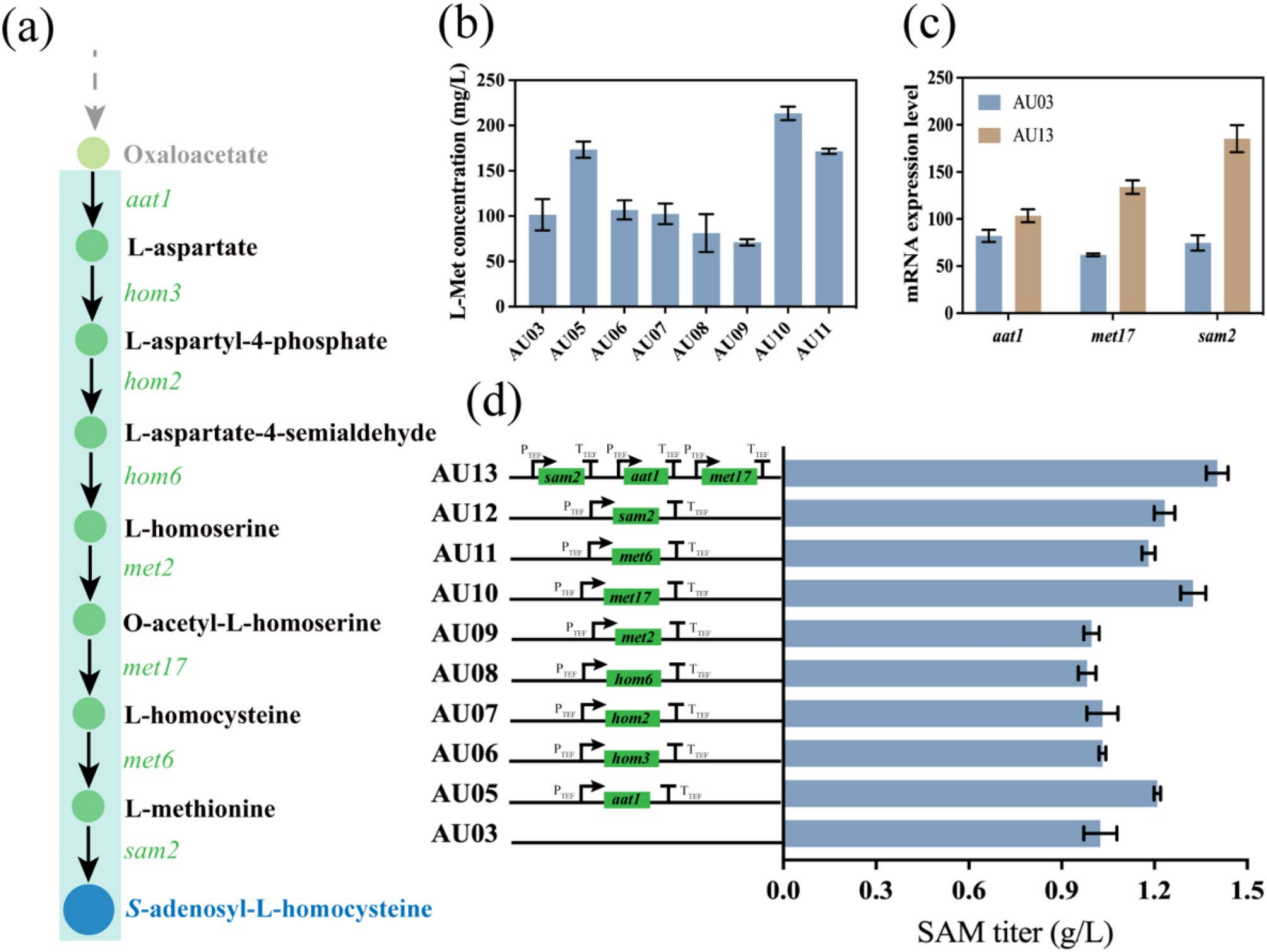
Effect of Overexpressing *aat1, met17*, and *sam2* on SAM production of mutants. (**a**) Biosynthetic pathway of SAM. (**b**) Intracellular L-Met concentration in mutants. (**c**) The transcript levels of *aat1, met17*, and *sam2* genes in mutants. (**d**) SAM titer of mutants in shaking flask fermentation

In *S. cerevisiae*, two enzymes are responsible for SAM synthesis, encoded by the *sam1* and *sam2* genes. The enzyme encoded by *sam1* is inhibited by excess L-Met, whereas *sam2* is not (Kodaki et al. 2003). To enhance SAM production, the *sam2* gene was overexpressed in mutant AU02, generating a mutant AU12. In addition, to further investigate the key rate limiting enzymes in the SAM biosynthesis pathway, genes involved in the TCA cycle to L-Met synthesis include *aat1, hom3, hom2, hom6, met2, met17*, and *met6* were also overexpressed (Fig. 4a). This resulted in the mutants AU05, AU06, AU07, AU08, AU09, AU10, and AU11. After culturing these mutants in YPD for 12 h, their intracellular L-Met levels were measured (Fig. 4b). The results showed that overexpression of *aat1, met17*, and *met6* significantly increased L-Met synthesis. Shake flask fermentation was further performed to evaluate the SAM production of these mutants. The results demonstrated that overexpressing *aat1, met17, met6*, and *sam2* significantly increased SAM titer, reached 1.21 g/L, 1.33 g/L, 1.18 g/L, and 1.23 g/L, respectively, resulting in a 17.48%, 29.13%, 14.56%, and 19.42% increase compared to mutant AU02 (Fig. 4d). Subsequently, the genes for these key enzymes were combined, and the optimal combination (overexpressing *aat1, met17*, and *sam2*) was integrated into the genome, resulting in mutant AU13. Following successful gene insertion, real-time fluorescence quantitative PCR was performed on mutant AU13 to measure the expression levels of the overexpressed genes. As shown in Fig. 4c, the expression levels of all overexpressed genes were significantly elevated. Shake flask fermentation of mutant AU13 was then carried out. Obviously, after enhancing the supply of precursor L-Met and strengthening the synthesis of SAM, the SAM titer significantly increased to 1.4 g/L (Fig. 4d), resulting into a 35.92% increase compared to mutant AU03. The results indicate that, consistent with previous studies (Chen et al. 2016a; Wang et al. 2024b; Xiao et al. 2024), SAM synthetase is a key limiting enzyme in SAM synthesis. Overexpression of the *sam2* gene effectively enhanced SAM production and resulted in a significant increase in SAM production. Furthermore, mitochondrial aspartate aminotransferase (encoded by *aat1*) is also a rate-limiting enzyme in the SAM biosynthesis pathway which catalyzes the conversion of oxaloacetate to aspartate (Morin et al. 1992). Overexpression of this enzyme can increase the conversion of oxaloacetate to aspartate in the TCA cycle and thereby redirect more carbon flux towards SAM production. Additionally, the conversion of O-acetyl-L-homoserine and hydrogen sulfide to L-homocysteine represents another key rate-limiting node in SAM biosynthesis. The O-acetylhomoserine (thiol)-lyase (encoded by *met17*) plays a crucial role in L-Met and cysteine biosynthesis and inorganic sulfur assimilation (Brzywczy and Paszewski 1993; Yamagata et al. 1994). Therefore, overexpression of this enzyme effectively alleviated this bottleneck and thereby enhanced SAM production.

### Enhancing supply of precursors

**Fig. 5:**
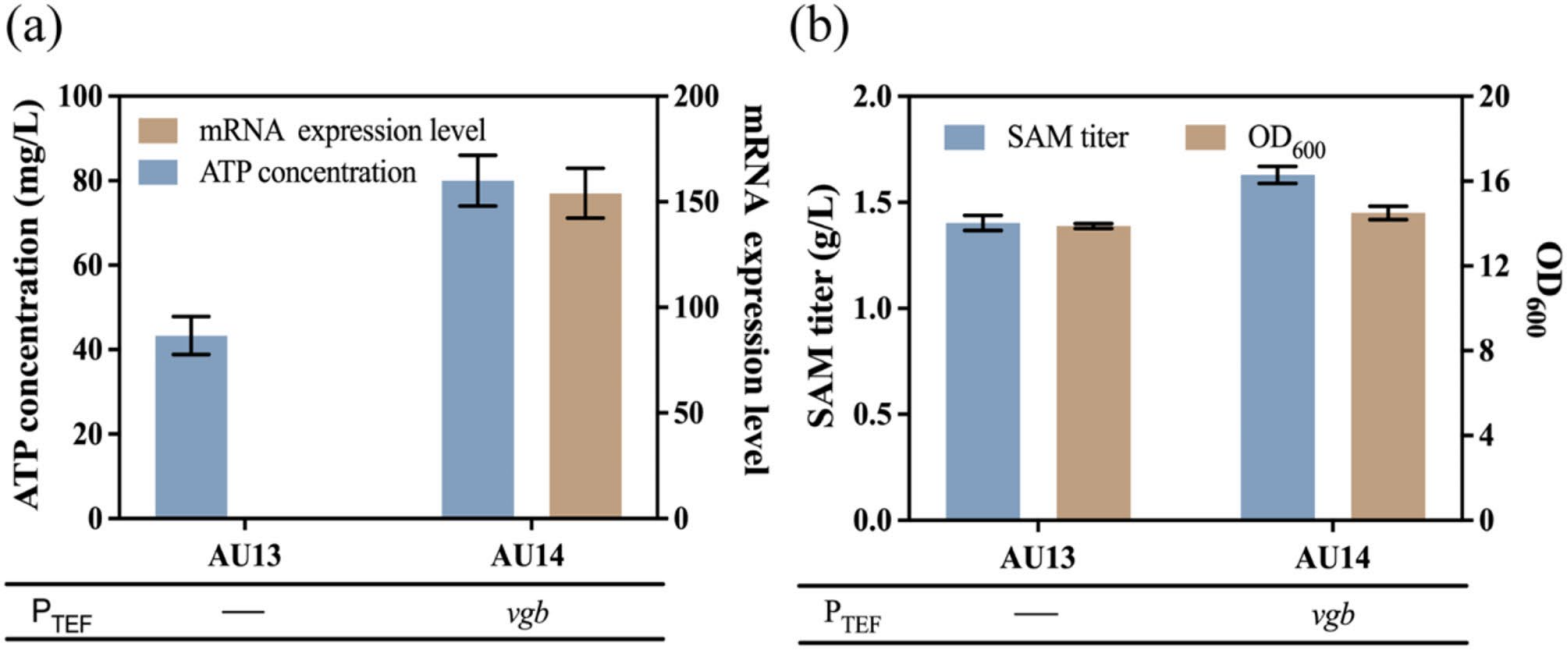
The effect of expression *vgb* gene on ATP and SAM titer of the mutants. (**a**) The ATP concentration and mRNA expression level of mutant expressing the *vgb* gene. (**b**) The SAM titer and OD_600_ of mutant expressing *vgb* gene

Ensuring an adequate supply of precursor is critical for enhancing product titer (Yang et al. 2020). As one of the precursors for SAM synthesis, ATP not only serves as both a precursor but also an energy source for SAM synthesis. Therefore, increasing ATP availability is essential for improving SAM production. VHb, enables *Pseudomonas aeruginosa* to thrive under microaerobic conditions. This protein modulates cellular metabolism in oxygen-limited environments and promotes cell growth and protein synthesis (Stark et al. 2012). It is hypothesized that introducing *vgb* could alleviate this limitation by increasing intracellular oxygen levels and ultimately improving SAM titer. To verify this hypothesis, the *vgb* gene was introduced into mutant AU13, resulting in mutant AU14. The relative expression level of *vgb* in mutant AU14 was assessed by real-time quantitative PCR (Fig. 5a). The intracellular ATP levels were also measured. The results indicated the successful expression of *vgb* in mutant AU14 with ATP concentrations reaching 80 mg/L (Fig. 5a), resulting in an 84.76% increase compared to mutant AU13. To further investigate the influence of *vgb* expression on SAM production, shake flask fermentation was performed. The SAM titer of mutant AU14 reached 1.63 g/L, resulting in a 19.29% increase compared to mutant AU13 (Fig. 5b). The increase in ATP content following *vgb* overexpression is likely due to VHb providing storage site for oxygen and facilitates its transfer to the terminal respiratory oxidase, and thereby enhances ATP production, reduces NADH levels, and increases the cell membrane potential (Webster et al. 2021). The resulting over accumulation of ATP further promotes SAM synthesis. With this study and previous research (Chen and Tan 2018), it has been demonstrated that ATP is one of the bottlenecking factors in SAM synthesis, and improving the supply of ATP can effectively enhance SAM production. As another precursor, L-Met, can be transported into *S. cerevisiae* via two methionine permeases encoded by the *mup1* and *mup3* genes (Isnard et al. 1996). In this research, to verify whether methionine permeases are a limiting factor for SAM synthesis, the *mup1* and *mup3* genes were overexpressed in mutant AU14 using the pESC-TEF overexpression plasmid. As shown in Fig. S3, the SAM titers of strains overexpressing *mup1* and *mup3* reached 1.65 and 1.60 g/L, respectively. There is no significant difference between the engineered strains and mutant AU14. This indicates that methionine permease is not a limiting factor for SAM production in the AU14 strain which is consistent with the findings of Ravi et al. (2014).

### Blocking the catabolic pathway of SAM

In *S. cerevisiae*, two distinct metabolic pathways are involved in metabolism of SAM: (1) SAM is converted to SAH by SAM: C-24 sterol methyltransferase (encoded by *erg6*) (Qu et al. 2019). SAH is subsequently hydrolyzed by *S*-adenosyl-L-homocysteine hydrolase (encoded by *sah1* gene), producing L-homocysteine and thereby completing the SAM cycle (Visram et al. 2018). (2) SAM is decarboxylated by S-adenosylmethionine decarboxylase (encoded by *spe2* gene) to form S-adenosyl 3-propylamine. This intermediate is further metabolized into L-Met, polyamines, and pantothenic acid (Balasundaram et al. 1994). Therefore, theoretically, knocking out the *erg6, sah1* and *spe2* genes could promote SAM accumulation.

To investigate the effect of blocking SAM catabolic pathway on SAM accumulation, the *erg6, sah1*, and *spe2* genes were deleted in mutant AU14, resulting in mutant AU15, AU16, and AU17 (Fig. 3b). The fermentation performance of mutant AU14, AU15, AU16, and AU17 was evaluated in shake flask. As shown in Fig. 3d, except for mutant AU15, all other mutants with downregulated genes exhibited increased SAM titer. Specifically, mutant AU16 produced 1.73 g/L SAM, while mutant AU17 produced 1.77 g/L SAM. Subsequently, the *spe2* gene was knocked out in mutant AU16, resulting in mutant AU18. Although biomass significantly decreased, the SAM titer of mutant AU18 reached 1.86 g/L, representing a 14.11% increase compared to mutant AU14. The differences in SAM titers among these mutants may be ascribed to: (1) Knockout of the *erg6* gene in the first SAM metabolic pathway not only reduced the SAM titer but also significantly affected strain growth, likely due to the disruption of ergosterol biosynthesis. Ergosterol is a major sterol in fungal membranes and plays a crucial role in maintaining membrane fluidity and regulating various cellular processes (Choy et al. 2023). (2) In contrast, knocking out the *sah1* gene in this pathway did not affect strain growth, but significantly increased SAM titer. This is likely because deletion of the *sah1* gene does not directly disrupt the methylation reaction itself, but instead blocks the further metabolism of SAH, the product of SAM demethylation. SAH acts as a competitive inhibitor in the transmethylation reaction (Tehlivets et al. 2004), and when it accumulates to a certain level, it can inhibit the activity of methyltransferases, thereby promoting SAM accumulation. Therefore, knocking out the *sah1* gene prevents the degradation of SAM and enhances its accumulation without affecting normal strain growth. (3) Although the deletion of *spe2* has been reported to cause a deficiency in spermidine (Chattopadhyay et al. 2006), as shown in Fig. 3d, no significant differences in growth were observed between AU17, AU18, and AU14. As previously reported (Zhao et al. 2016), spermidine deficiency does not affect the normal strain growth, likely due to trace amounts of spermidine derived from yeast extract, which are sufficient to meet the growth requirements of the host. These results suggest that appropriately blocking the further metabolic pathways of SAM is also a promising strategy for developing high SAM-producing yeast strains.

### Fermentation optimization

#### Shake flask optimization

**Fig. 6:**
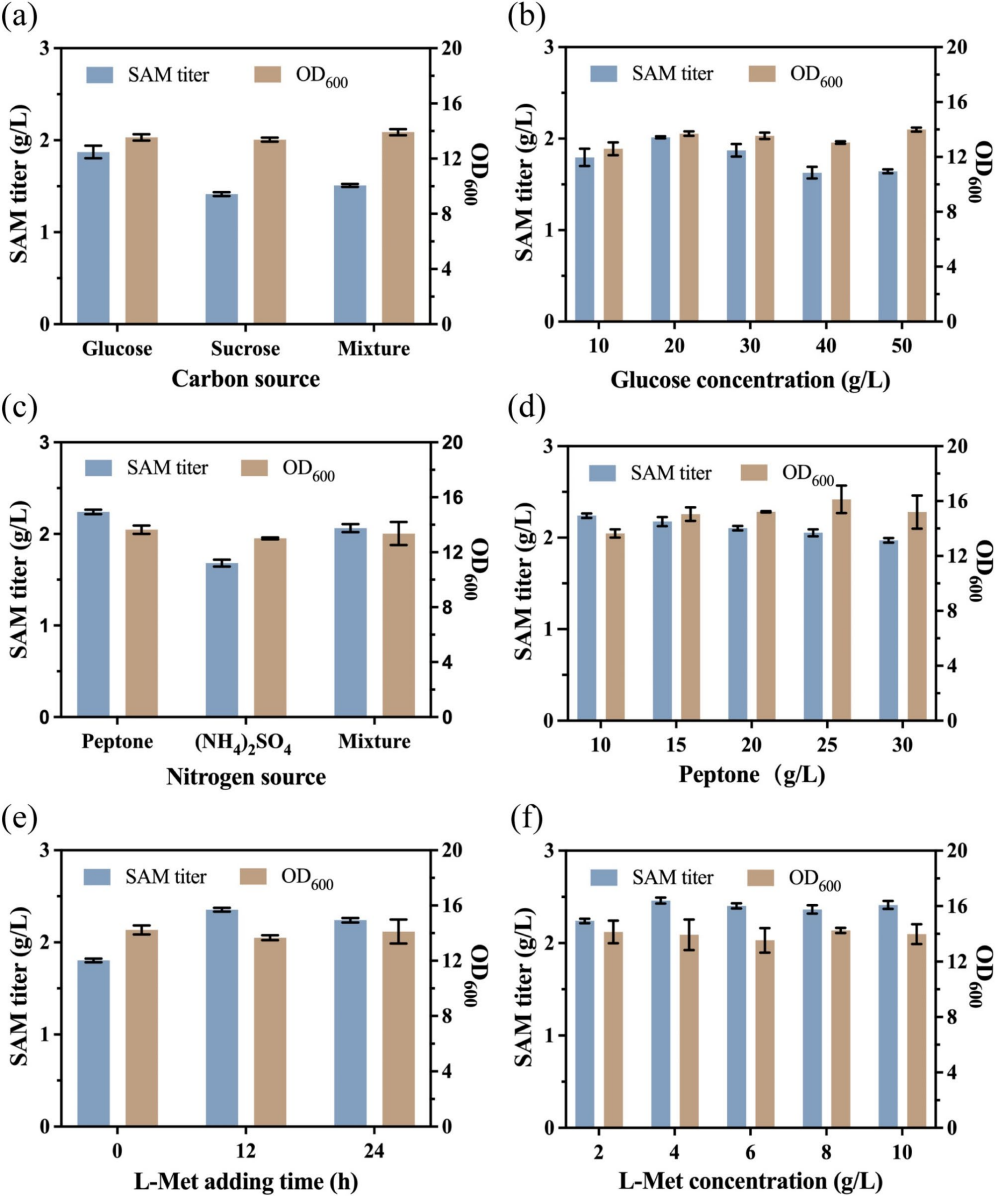
The effect of fermentation media and feeding on SAM titer and growth of mutant AU18. (**a**) The effect of carbon source concentrations. (**b**) The effect of glucose concentrations. (**c**) The effect of nitrogen source concentrations. (**d**) The effect of peptone concentrations. (**e**) The effect of L-Met adding time. (**f**) The effect of L-Met concentrations. The “Mixture” refers to the blend of the remaining two components in a 1:1 ratio in the same experiment

The carbon source, nitrogen source, and yeast extract in the medium are critical for yeast growth and SAM formation. Additionally, the addition of L-Met is essential for SAM production during fermentation, as high concentrations of L-Met can inhibit yeast growth, while low concentrations of L-Met are insufficient for SAM synthesis. Therefore, in this study, a series of optimizations were performed using a single-factor strategy to adjust the types and concentrations of carbon and nitrogen sources, yeast extract, and L-Met feeding strategy in the fermentation medium. Specifically, carbon sources are not only essential components of yeast cells but also provide energy for yeast cell growth and regulate cellular metabolism. Therefore, carbon sources are indispensable nutrients for the growth of *S. cerevisiae* and SAM synthesis. In studies on SAM production using *S. cerevisiae*, glucose or sucrose is commonly used as the carbon source (Hu et al. 2023; Wang et al. 2024b; Weng et al. 2022; Xiao et al. 2024). Therefore, glucose and sucrose were selected for the exploration of the optimal carbon source for AU18 in this study. From Fig. 6a and b, it can be seen that using glucose as the sole carbon source and 20 g/L glucose was optimal for SAM production in mutant AU18. This supports the notion that the *hxk2* gene enhances glucose uptake into the cell, eliminating the need for high concentrations of glucose in the medium. Nitrogen sources are another essential component for the growth and SAM synthesis of *S. cerevisiae*. In previous studies (Hu et al. 2023; Qin et al. 2020; Weng et al. 2022; Xiao et al. 2024), peptone or (NH_4_)_2_SO_4_ has typically been used as the nitrogen source. Therefore, in this study, the optimal type and concentration of nitrogen source for AU18 were explored. The data (Fig. 6c and d) revealed that the highest SAM titer was obtained when peptone was used as the sole nitrogen source at 25 g/L. The main components of yeast extract include peptides, amino acids, and flavor nucleotides, all of which play a crucial role during fermentation. Yeast extract not only provides essential nutrients but also improves fermentation quality, simplifies the extraction process, and enhances overall efficiency (Tao et al. 2023). Therefore, yeast extract is a fundamental component of the fermentation medium for *S. cerevisiae* (Hu et al. 2023; Qin et al. 2020; Wang et al. 2024b; Weng et al. 2022; Xiao et al. 2024). Thus, in this research, the optimal concentration of yeast extract was investigated. From Fig. S4, it can be seen that although higher yeast extract concentrations led to increased biomass, no further improvement in SAM titer was observed when yeast extract exceeded 10 g/L. L-Met, as one of the precursors for SAM synthesis, is an essential component for SAM biosynthesis. It is typically added during fermentation to meet the demand for SAM production. However, excessive L-Met can suppress the growth of yeast (Kodaki et al. 2003). Therefore, to further improve the SAM titer, the supplementation time and concentration of L-Met were explored. The results showed that supplementation of L-Met at 12 h, during the rapid growth phase, enhanced SAM synthesis (Fig. 6e), with the highest SAM titer obtained at 4 g/L L-Met (Fig. 6f). Under the optimal medium and feeding strategy, the SAM titer and OD_600_ of mutant AU18 in shake flask fermentation reached 2.46 g/L and 13.93, respectively. The optimal medium composition was determined to be 20 g/L sucrose, 10 g/L yeast extract,25 g/L peptone, 4 g/L KH_2_PO_4_, 2 g/L K_2_HPO_4_, and 0.5 g/L MgSO_4_·H_2_O.

### 5 L fermenter fermentation

**Fig. 7:**
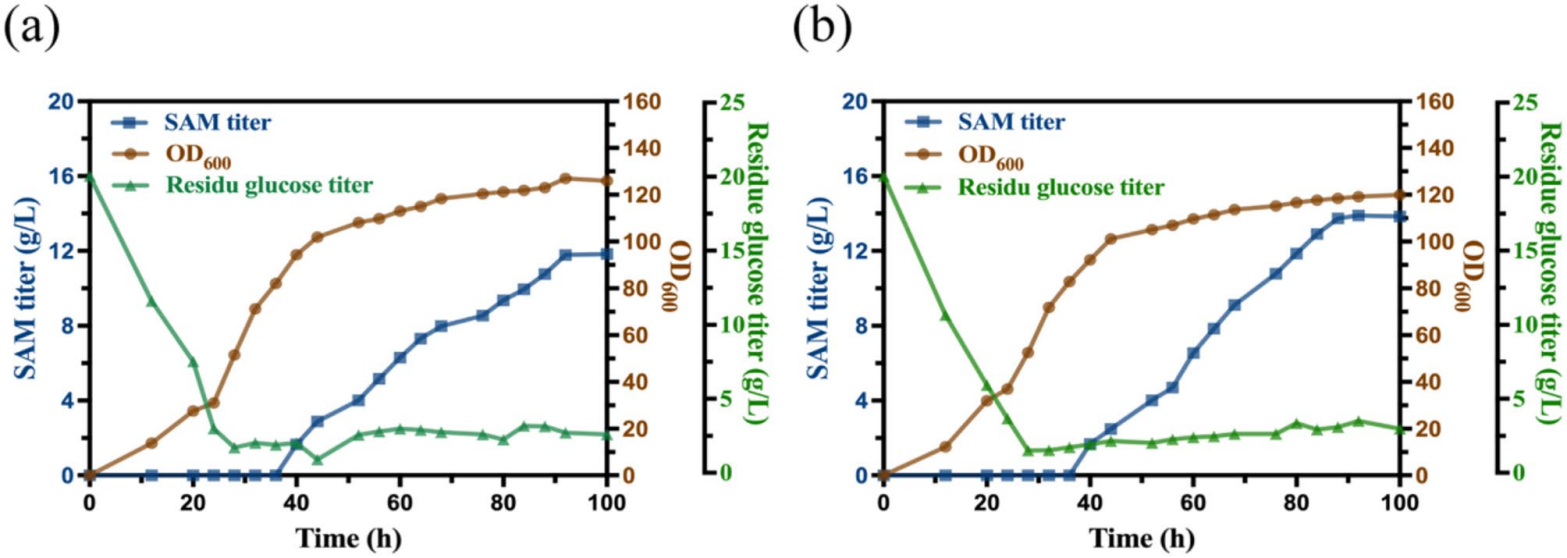
Time course of SAM production and growth of mutant AU18 in 5 L fermenter with different L-Met feeding strategies. (**a**) Intermittent L-Met feeding strategy. (**b**) Continuous L-Met feeding strategy

The fermentation was scaled up in a 5 L fermenter to further validate the SAM production of mutant AU18. Regulation of L-Met concentration during fermentation in the 5 L fermenter is critical. To maintain a relatively stable L-Met concentration and minimize fluctuations, the strategy of L-Met addition was improved based on previous study (Weng et al. 2022) and employed a continuous feeding strategy to reduce fluctuations as much as possible. To evaluate the effect of L-Met feeding strategy on SAM synthesis, two strategies were examined: (1) Intermittent feeding: in which 8 g L-Met was added every 4 h, and (2) Continuous feeding: in which 50 g/L L-Met was added at a rate of 0.5 mL/min. As shown in Fig. 7, continuous feeding of L-Met resulted in a maximum SAM titer of 13.96 g/L at 92 h, whereas intermittent feeding achieved a maximum titer of 11.53 g/L at 100 h. These results indicated that continuous feeding of L-Met is more beneficial for the synthesis of SAM, not only achieving higher titer, but also shortening the fermentation time, meanwhile reduces the amount of L-Met supplementation. This is because intermittent feeding may cause a decrease in L-Met levels during the final phase of each 4 h period, potentially insufficient for SAM synthesis. The continuous feeding of L-Met addresses this issue by maintaining an excess of L-Met in the fermentation broth, enables continuous SAM synthesis and reduces its degradation. However, the excess L-Met inhibited strain growth and leading to slower biomass formation.

## Conclusion

The growing demand for SAM underscores the need for more efficient production methods. In this study, the growth characteristics of the strain AU were first optimized, followed by a comprehensive optimization of its SAM biosynthetic pathway using a modular strategy. This approach involved enhancing the supply of precursors, such as L-Met and ATP, while minimizing the degradation of SAM. The final mutant, AU18, achieved a SAM titer of 1.78 g/L in shake flask fermentation, resulting in a 227.67% increase compared to the parental strain AU. In this study, the overexpression of the *hxk2* and *aat1* genes was firstly employed to promote glucose metabolism and to competitively divert more oxaloacetate from the TCA cycle, thereby enhancing SAM synthesis. Additionally, a comprehensive analysis and optimization of the synthetic and degradation pathways of SAM were conducted. The composition of the fermentation medium was further optimized to meet the nutritional requirements of mutant AU18. With the optimal medium, the SAM titer reached 2.46 g/L. Moreover, various L-Met feeding strategies for SAM production of mutant AU18 were examined in a 5 L fermenter. The continuous feeding of L-Met resulted in a SAM titer of 13.89 g/L, which is relatively high among the reported yields so far (Table 1). Therefore, in this research the SAM biosynthesis of *S. cerevisiae* was significantly enhanced with the multimodule strategy based on comprehensive optimization of both synthesis and degradation pathways of SAM. The established multimodule strategy study provides a foundation for the development of *S. cerevisiae* with improved productivity of SAM and other target metabolites.

## Electronic supplementary material

Below is the link to the electronic supplementary material.

Supplementary Material 1

## Data Availability

All data generated during this study are included in this published article.

## Abbreviations

SAM: S-adenosyl-L-methionine
SAH: *S*-adenosyl-L-homocysteine
GYC: Glyoxylate cycle
UV: Ultraviolet
ARTP: Atmospheric and room temperature plasma
TCA: Tricarboxylic acid
YPD: Yeast peptone dextrose
LB: Luria–Bertani
HPLC: High-performance liquid chromatography
VHb: *Vitreoscilla* Hemoglobin
DO: Dissolved oxygen
