# Supplementary material for: System metabolic engineering modification of Saccharomyces cerevisiae to increase SAM production
Source: Bioresour Bioprocess. 2025 Mar 12;12(1):19. doi: 10.1186/s40643-025-00858-9 (PMC11904041; doi:10.1186/s40643-025-00858-9)
Supplement: Supplementary file 1 — Supplementary Material 1 [file 40643_2025_858_MOESM1_ESM.docx]

**System metabolic engineering modification of *Saccharomyces cerevisiae* to increase SAM production**

^1^Key Laboratory of Bioorganic Synthesis of Zhejiang Province, College of Biotechnology and Bioengineering, Zhejiang University of Technology, No. 18, Chaowang Road, Hangzhou, Zhejiang Province 310014, P. R. China

^2^Engineering Research Centre of Bioconversion and Biopurification, Ministry of Education, Zhejiang University of Technology, No. 18, Chaowang Road, Hangzhou, Zhejiang Province 310014, P. R. China

^3^The National and Local Joint Engineering Research Centre for Biomanufacturing of Chiral Chemicals，Zhejiang University of Technology, Hangzhou 310014, P. R. China

***Correspondence author:**

Yuanshan Wang, Tel: +86-571-88320391, Fax: +86-571-88320630, Email: yuanshan@zjut.edu.cn


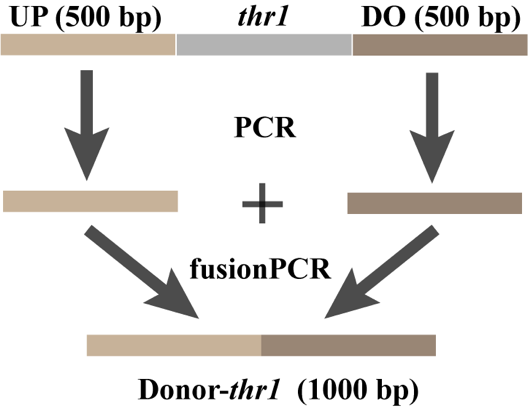


**Fig. S1** The construction process of the donor DNA.


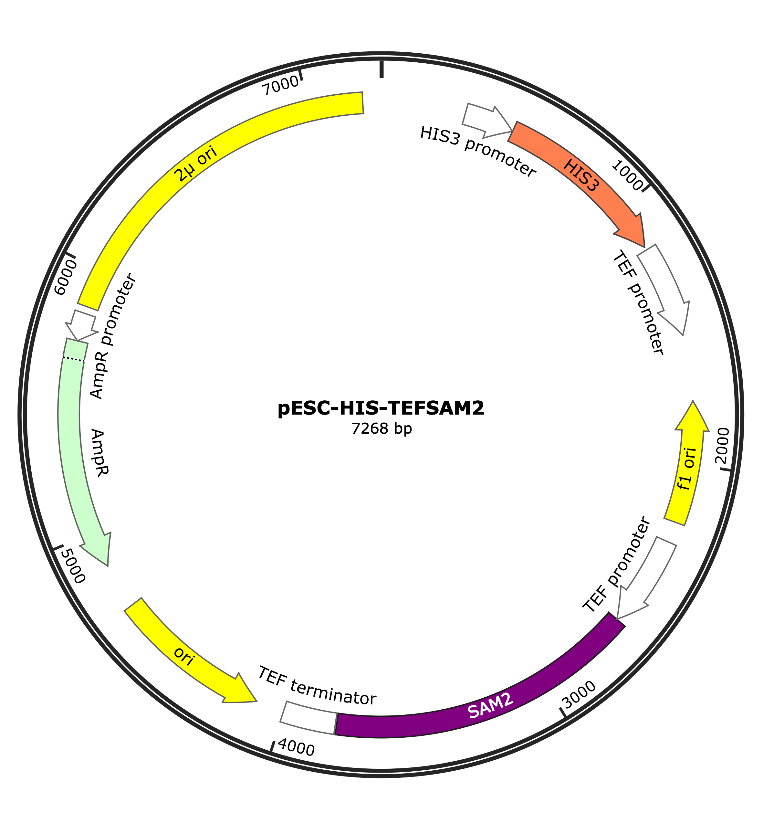


**Fig. S2** The *sam2* overexpressing plasmid.


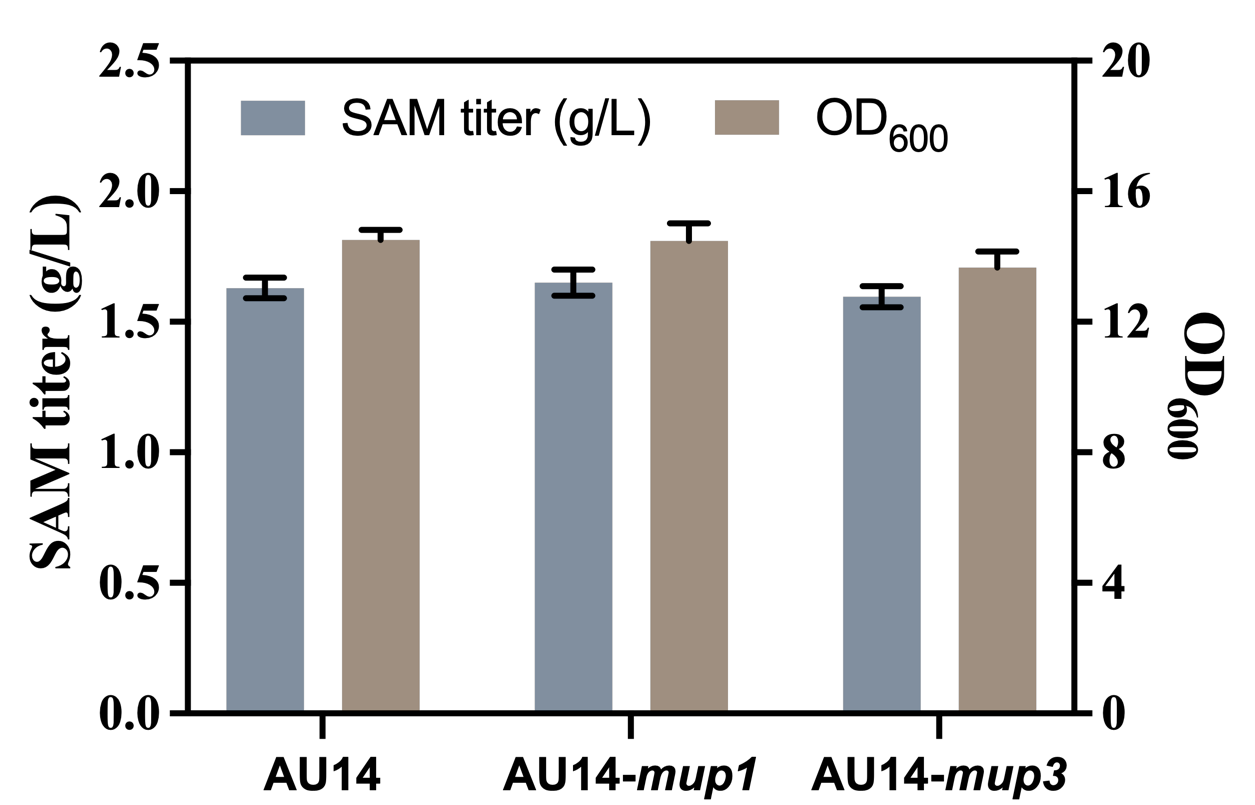


**Fig. S3** The effect of expressing *mup1* and *mup3* genes on SAM titer and growth of mutant AU14.


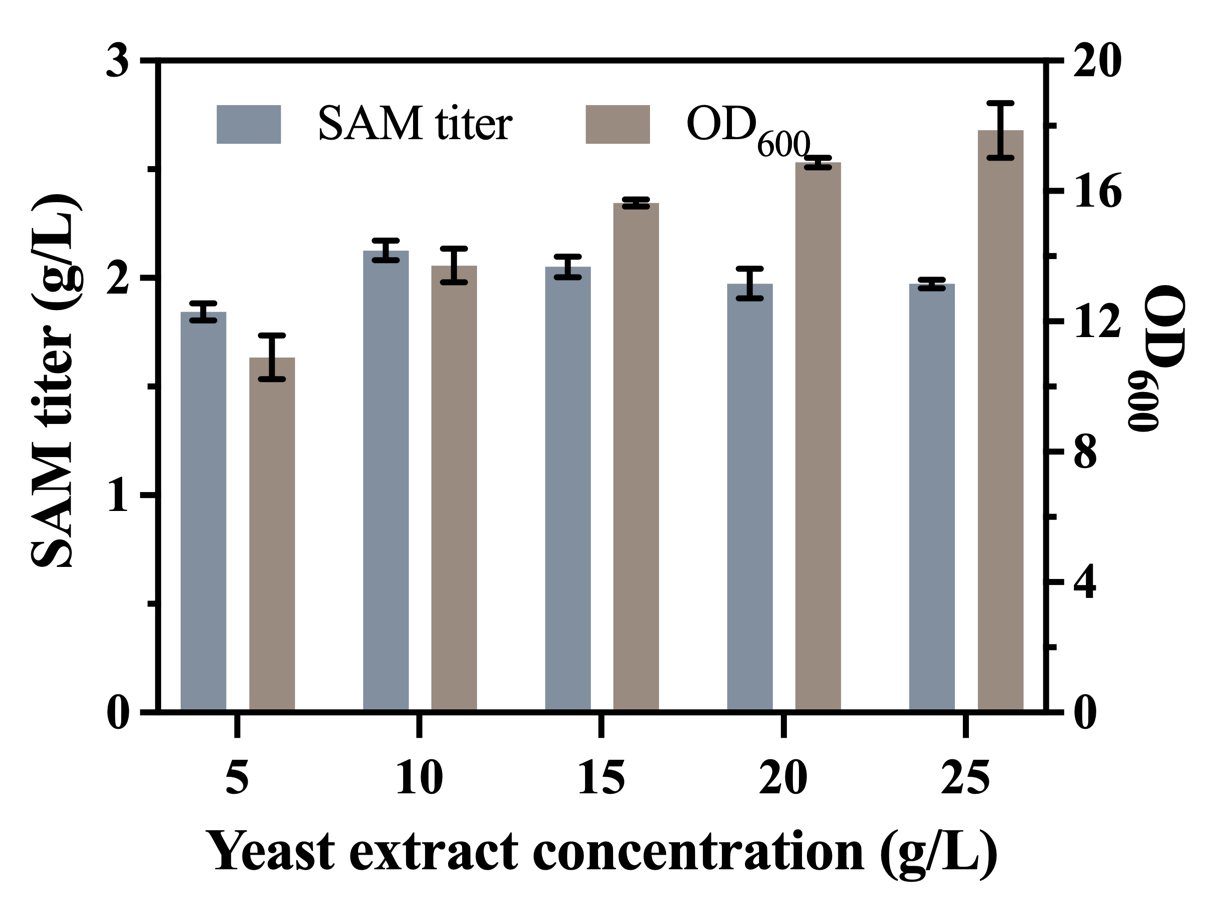


**Fig. S4** The effect of yeast extract concentrations on SAM titer and growth of mutant AU18.

**Table S1** Primers used in this study.

| Primers | Sequence (5ʹ-3ʹ) |
| --- | --- |
| thr1-up-f | ctccaacgtctctgccattg |
| thr1-up-r | ggggtaaaggacatttttctgtagtccgtatatctactttatgctac |
| thr1-do-f | cggactacagaaaaatgtcctttacccctagtatactgtg |
| thr1-do-r | aggggatgtccttaacaagaacttc |
| ura4-up-f | aatcatgagactaatagaacaacactaggg |
| ura4-up-r | ctacatattgttgcatatgctttgttaggtattacttaagtgatatagtg |
| ura4-do-f | gtaatacctaacaaagcatatgcaacaatatgtaggctcatttg |
| ura4-do-r | ttgatcctatttctttgattttaattcctggag |
| cys4-up-f | gtgttctcatccgaccctctg |
| cys4-up-r | gaagcgtgggttcttatttttattcttacgtcgtatttatctagatgtcc |
| cys4-do-f | cgacgtaagaataaaaataagaacccacgcttcaaataaaag |
| cys4-do-r | tcttcttctgggatctgtcatc |
| pESC-TEF-aat1-F | gtcaaggagaaaaaacatgttgaggacgaggcttactaattg |
| pESC-TEF-aat1-R | gtattacggatccgggttatgcgagtttgtccattttcgag |
| pESC-TEF-aat2-F | cgtcaaggagaaaaaacatgtctgccactctgttcaataacatc |
| pESC-TEF-aat2-R | gtattacggatccgggttacaatttagcttcaatagtatagaagcgcac |
| pESC-TEF-hom2-F | cgtcaaggagaaaaaacatggctggaaagaaaattgctgg |
| pESC-TEF-hom2-R | gtattacggatccgggttaaatcaagtttcttgctagtaagatttcggc |
| pESC-TEF-hom3-F | gtcaaggagaaaaaacatgccaatggatttccaacctac |
| pESC-TEF-hom3-R | gtattacggatccgggttaaattccaagtcttttcaattgttctaaacg |
| pESC-TEF-hom6-F | gtcaaggagaaaaaacatgagcactaaagttgttaatgttgc |
| pESC-TEF-hom6-R | gtattacggatccgggctaaagtctttgagcaatcttgataacatcac |
| pESC-TEF-met2-F | gtcaaggagaaaaaacatgtcgcatactttaaaatcgaaaacgc |
| pESC-TEF-met2-R | gtattacggatccgggctaccagttggtaacttcttcggc |
| pESC-TEF-met6-F | gtcaaggagaaaaaacatggttcaatctgctgtcttaggg |
| pESC-TEF-met6-R | gtattacggatccgggttaattcttgtattgttcacggaagtacttg |
| pESC-TEF-met17-F | gtcaaggagaaaaaacatgccatctcatttcgatactgttc |
| pESC-TEF-met17-R | gtcgtattacggatccgggtcatggtttttggccagcg |
| YZ-pESC-F | cttctctttggaactttcagtaatacgc |
| YZ-pESC-R | gttcaccatcaccgtaagtagc |
| pESC-(BDK)-F | catacgagccggaagcataaagctggcgtttttccataggctc |
| pESC-(BDK)-R | ctggcgttacccaacttaacagctggcgtaatagcgaagag |
| pESC-TEF-vgb-F | aacatacgagccggaagcataaag |
| pESC-TEF-vgb-R | gtgtggaattgtgagcggat |
| pESC-TEF-sam2-F | gaaataatttcacacaggaaacagaccatgtccaagagcaaaactttcttatttac |
| pESC-TEF-sam2-R | gagtcgtattacggatccgggttaaaattccaatttctttggtttttc |
| qaat1-f | ttatggtgaacgagttggt |
| qaat1-r | cctggtggagaagaatacat |
| qvgb-f | cattaccaccaccttctaca |
| qvgb-r | ttcacggcaatcttcttca |
| q-met 17-f | acttggcatacatcgttca |
| q-met 17-r | actaaggtcttggcatcac |
| q-sam2-f | cacgaactatcagccactt |
| q-sam2-r | cacgataactcttgctcaac |
| q-act1-f | atcctacgaacttccagatg |
| q-act1-r | gccaagatagaaccaccaa |
